# Predictors Associated with Adverse Pregnancy Outcomes in a Cohort of Women with Systematic Lupus Erythematosus from Romania—An Observational Study (Stage 2)

**DOI:** 10.3390/jcm11071964

**Published:** 2022-04-01

**Authors:** Petronela Vicoveanu, Ingrid-Andrada Vasilache, Dragos Nemescu, Alexandru Carauleanu, Ioana-Sadiye Scripcariu, Dorina Rudisteanu, Alexandra Burlui, Elena Rezus, Demetra Socolov

**Affiliations:** 1 Department of Obstetrics and Gynecology, ‘Grigore T. Popa’ University of Medicine and Pharmacy, 700115 Iasi, Romania; petronelapintilie@yahoo.com (P.V.); dnemescu@yahoo.com (D.N.); acarauleanu@yahoo.com (A.C.); isscripcariu@gmail.com (I.-S.S.); rudisteanu.dorina@yahoo.com (D.R.); demetrasocolov@gmail.com (D.S.); 2 Department of Rheumatology and Rehabilitation, ‘Grigore T. Popa’ University of Medicine and Pharmacy, 700115 Iasi, Romania; alexandra.burlui@gmail.com (A.B.); elena_rezus@yahoo.com (E.R.)

**Keywords:** predictors, adverse pregnancy outcomes, systemic lupus erythematosus

## Abstract

Background: Pregnancy in women with systemic lupus erythematosus (SLE) is accompanied by adverse pregnancy outcomes (APOs). We aimed to investigate the association between clinical, sonographic, and laboratory parameters and APOs (preeclampsia, intrauterine growth restriction, premature birth, and maternal mortality). Methods: This observational retrospective study included all pregnancies in women with SLE who attended two tertiary maternity hospitals from Romania between January 2013 and December 2020. Clinical, sonographic, and laboratory variables were examined. Bivariate associations of APO status and each predictor variable were evaluated, and significant predictors were further included in a classification model based on discriminant analysis. Results: Predictors of APOs included BMI > 25 kg/m^2^, personal history of lupus nephritis or chronic hypertension, proteinuria, low C3, SLE Disease Activity Index 2000 (SLEDAI-2k score ≥ 4 and physician’s global-assessment (PGA) score ≥ 1 throughout pregnancy, increased mean uterine arteries pulsatility index in the first and second trimesters, cerebroplacental ratio < 1 in the second and third trimesters, and small fetal abdominal circumference in the third trimester. Glucocorticoids, methyldopa, and aspirin use appeared to be protective against APOs. Conclusions: This study provides a comprehensive analysis of the most important predictors for APOs in pregnant patients with SLE, which could constitute a basis for further research.

## 1. Introduction

Systematic lupus erythematosus (SLE) is a chronic autoimmune disease characterized by multiple organ systems impairment, and is associated with serious long-term complications. Pregnancy for women with SLE can be encumbered by numerous severe adverse pregnancy outcomes (APOs), such as prematurity, intrauterine growth restriction (IUGR), preeclampsia (PE), fetal loss, and maternal morbidity and mortality [1,2]. Nevertheless, it has been stated that rigorous evaluations and pregnancy planning could reduce the frequency of APOs in women with SLE. In this respect, disease activity at the moment of conception is crucial; women with mild disease manifestations or in remission demonstrate notably better pregnancy outcomes [3,4].

The incidence of SLE ranges between 0.3 and 31.5 cases per 100,000 individuals per year, with a tendency toward growth in recent decades, and has a striking female predominance [5]. Prevalence rates worldwide are approximately 50–100 per 100,000 adults [5]. For the Caucasian population, it is estimated that most patients are middle-aged women, and approximately 50% of SLE cases are mild at presentation [5]. Nevertheless, the onset of SLE in women occurs predominantly during childbearing age, partly due to the involvement of hormonal factors in the pathogenesis of the disease [6,7].

The diagnosis of SLE is clinical, supported by laboratory findings. The combination of all three sets—ACR-1997 (American College of Rheumatology 1997), SLICC-2012 (Systemic Lupus International Collaborating Clinics 2012), and EULAR (European League Against Rheumatism)/ACR-2019)—ensures the capture of non-overlapping groups of patients [8,9,10]. The SLICC-2012 and EULAR/ACR-2019 criteria, but not the ACR-1997 criteria, necessitate the presence of antinuclear antibodies (ANAs) or other immunologic elements (autoantibodies or hypocomplementemia).

The SLICC and EULAR/ACR criteria are more sensitive than the ACR criteria in patients with early illness, and the EULAR/ACR criteria have greater specificity [11]. More changes to the classification criteria could improve their sensitivity, allowing for the early diagnosis and treatment of more SLE patients.

Several studies have investigated numerous clinical and laboratory predictors for adverse pregnancy outcomes in patients diagnosed with SLE, but the phenotypical polymorphism of the disease represents a challenge for obstetricians. Among the studied APO predictors, high clinical SLE disease activity at baseline, a history of lupus nephritis or active nephritis at conception, the presence of lupus anticoagulant, nonwhite ethnicity, the use of antihypertensive medications at baseline, thrombocytopenia, and aberrant activation of the alternate complement pathway had a significant impact on the evolution of pregnancy [12,13,14].

The identification of clinical and laboratory parameters that predict adverse pregnancy outcomes is vital for a better pre-conceptional counselling and SLE pregnancy management. Therefore, we conducted this study in order to investigate the association between clinical, sonographic, and laboratory parameters and APO in women with SLE.

## 2. Materials and Methods

We conducted an observational retrospective study of all pregnancies that occurred in women with SLE who attended two tertiary maternity hospitals: ‘Cuza -Voda’, Iasi, and ‘Saint John Emergency Hospital’, Suceava, Romania, between January 2013 and December 2020. Pregnant patients underwent follow-up at the rheumatology clinic from the Clinical Rehabilitation Hospital, Iasi. All patients fulfilled the SLICC-2012 (Systemic Lupus International Collaborating Clinics), the EULAR/ACR-2019 (European League Against Rheumatism/American College of Rheumatology), or the ACR 1997 classification criteria, depending on the date of SLE diagnosis. Ethical approval for this study was obtained from the Institutional Ethics Committees of University of Medicine and Pharmacy ‘Grigore T. Popa’ (No. 17806/3 September 2019), ‘Cuza -Voda’ Maternity Hospital, Iasi (No. 1254/1 February 2022), and ‘Saint John Emergency Hospital’, Suceava (No. 7/21 January 2022). Informed consent was obtained from all participants included in the study. All methods were carried out in accordance with relevant guidelines and regulations.

Medical records of patients were systematically reviewed, and data were obtained. Exclusion criteria comprised patients who had ectopic pregnancies, first and second trimester pregnancy loss, or patients that failed to participate in all the study visits. The following variables were recorded: demographic data, patient medical history, clinical SLE activity during pregnancy, BMI (body mass index), laboratory parameters (full blood count, complement 3 (C3), complement 4 (C4), proteinuria, hepatic enzymes, uric acid, urinary sediment analysis), autoantibodies which included antinuclear antibody (ANA), anti-double-stranded deoxyribonucleic acid (anti-dsDNA), anti-Ro antibody, anti-La antibody, lupus anticoagulant (LAC), anticardiolipin (aCL) and anti-β2 glycoprotein-I antibodies (aβ2GPI), sonographic elements (mean uterine arteries pulsatility index in the first and second trimesters, cerebroplacental ratio in the second and third trimesters, fetal abdominal circumference and venous duct aspect in the third trimester), use of medication, and pregnancy outcomes (Supplementary Material Tables S1 and S2).

The patients were followed over the entire course of their pregnancies. We considered adverse pregnancy outcomes the following entities: preeclampsia, IUGR, preterm birth, and maternal mortality. PE was defined as gestational hypertension with proteinuria or other maternal organ dysfunctions (maternal acute kidney injury, liver dysfunction, neurological features, hemolysis, or thrombocytopenia) at or after 20 weeks of pregnancy, with all symptoms disappearing by 12 weeks following delivery [15]. Maternal gestational hypertension was considered as systolic blood pressure ≥140 mmHg and/or diastolic blood pressure ≥90 mmHg measured on at least 2 occasions, 4 h apart, while a protein excretion of ≥300 mg/day in a 24 h urine collection was defined as proteinuria.

SGA was defined as the birth of a neonate with birth weight < 10th percentile according to Lubchenco growth curves for term infants and Fenton growth charts for premature infants [16,17]. All patients had sonography in the first trimester for pregnancy dating, and subsequent ultrasonographic evaluations were performed by experienced obstetricians, with at least level 2 qualification in ultrasound examination, using an E8 scanner with a 4.8 MHz transabdominal probe (GE Medical Systems, Milwaukee, WI, USA).

Preterm birth was considered the birth of a neonate before 37 completed weeks of gestation [18], and maternal mortality was defined as maternal death due to complications from pregnancy or childbirth [19]. Due to the small cohort size, pregnant patients were segregated into 2 groups: those with no adverse pregnancy outcomes, and those with at least 1 adverse pregnancy outcome. 

During all three trimesters of pregnancy, the SLE Disease Activity Index 2000 (SLEDAI-2k) was utilized to assess disease activity in individuals with SLE. The active phase of SLE was characterized as an SLEDAI-2k score of 4 or higher [20]. Physician’s global assessment (PGA) score (range 0 to 3, with 0 indicating inactive disease and 3 severe disease) was used to reflect the clinician’s judgement of overall SLE disease activity [21].

Statistical analysis was performed using SPSS software (version 28.0.1, IBM Corporation, Armonk, NY, USA). Bivariate associations of APO status and each predictor variable were evaluated with chi-square and Fisher’s exact tests for categorical variables, and *t*-test for continuous variables. A *p* value less than 0.05 was considered statistically significant. Variables with a significant *p* value from the univariate analysis were entered into a linear discriminant analysis using IBM SPSS Modeler (version 18.3).

## 3. Results

A total of 43 cases were screened for this study, but only 25 were included due to exclusion criteria and loss to follow-up. An SLE diagnosis was made for 20 patients, while 5 patients had an SLE diagnosis with positivity for specific antiphospholipid syndrome (APS) markers. All patients were Caucasian, with a mean age of 29.08 (±6.51 years SD). Several prenatal visits were requested during the first, second, and third trimesters of pregnancy for determination of clinical and biological status using the above-mentioned parameters. Characteristics of pregnant SLE patients in the first trimester and their association with adverse pregnancy outcomes are presented in Table 1.

During the first trimester, our results indicated a significant association between BMI > 25 kg/m^2^ (*p* < 0.001), personal history of lupus nephritis (*p* = 0.041) or chronic hypertension (*p* = 0.008), increased mean uterine arteries pulsatility index (*p* < 0.001), proteinuria (*p* = 0.004), low C3 (*p* = 0.004), SLEDAI-2k score ≥4 (*p* = 0.003), PGA score ≥1 (*p* < 0.001), and at least one adverse pregnancy outcome (preeclampsia, IUGR, prematurity, maternal death). Moreover, the patients who had at least one APO were found to be younger, with results approaching statistical significance (*p* = 0.054).

Characteristics of pregnant SLE patients in the second trimester, and their association with adverse pregnancy outcomes are presented in Table 2.

Hydroxychloroquine was recommended for 17 pregnant patients since the first trimester of pregnancy, and was continued postpartum for controlling disease activity and preventing flare-ups. Low-dose aspirin was initiated in 12 patients before 16 weeks of gestation, and was maintained until 36 weeks of gestation for prevention of preeclampsia in our cohort of patients. Methyldopa was the drug of choice for hypertension control during pregnancy, and was administered in 12 patients when needed (systolic blood pressure ≥140 mmHg and/or diastolic blood pressure ≥90 mmHg measured on at least 2 occasions, 4 h apart), regardless of the trimester of pregnancy, and was continued in the postpartum period if high blood pressure persisted.

Azathioprine at a maximum dose of 2 mg/kg/day was used in 4 patients in the second and third trimesters for controlling lupus nephritis manifestations. Pulse therapy with glucocorticoids was used for 14 patients to control SLE flares in the second and third trimesters of pregnancy, while low-dose glucocorticoids were used in 15 cases for maintenance therapy and for disease control beginning in the second trimester of pregnancy.

During the second trimester, our results indicated a significant association between increased mean uterine arteries pulsatility index (*p* < 0.001), cerebroplacental ratio less than 1 (*p* = 0.024), proteinuria (*p* = 0.032), low C3 (*p* < 0.001), SLEDAI-2k score ≥4 (*p* < 0.001), PGA score ≥1 (*p* < 0.001), and at least one adverse pregnancy outcome (preeclampsia, IUGR, prematurity, maternal death).

Characteristics of pregnant SLE patients in the third trimester, and their association with adverse pregnancy outcomes are presented in Table 3.

During the third trimester, our results indicated a significant association between a cerebroplacental ratio less than 1 (*p* = 0.024), small fetal abdominal circumference (*p* < 0.001), hepatic cytolysis (*p* = 0.024), proteinuria (*p* = 0.012), active urinary cast (*p* = 0.041), increased uric acid (*p* < 0.001), low C3 (*p* < 0.001), SLEDAI-2k score ≥4 (*p* = 0.014), PGA score ≥1 (*p* = 0.032), and at least one adverse pregnancy outcome (preeclampsia, IUGR, prematurity, maternal death).

**Figure 1:**
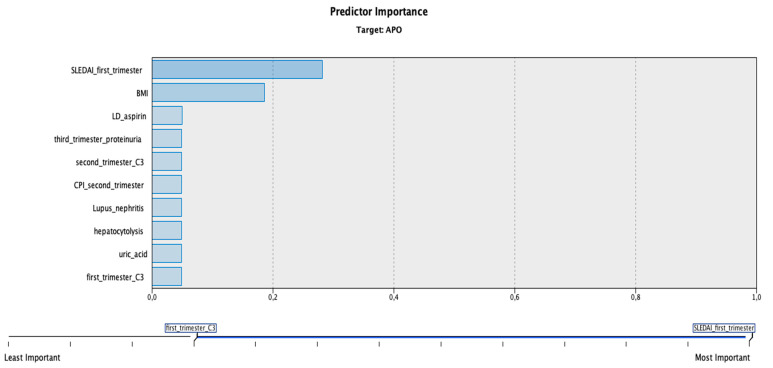
Importance of the predictors targeting adverse pregnancy outcomes according to the discriminant model. Legend: APO, adverse pregnancy outcome; SLEDAI_first_trimester, SLEDAI-2k score ≥4 from the first trimester; BMI, body mass index; LD_aspirin, use of low-dose aspirin; third_trimester_proteinuria, proteinuria present in the third trimester; second_trimester_C3, low complement (C3) in the second trimester; CPI_second_trimester, cerebroplacental index in the second trimester; Lupus_nephritis, personal history of lupus nephritis; hepatocytolysis, hepatic cytolysis; uric_acid, increased uric acid; first_trimester_C3, low complement (C3) level from the first trimester. Note: Commas represents the decimal separator.

All the significant parameters from the first, second, and third trimesters of pregnancy were included in a discriminant model using IBM SPSS Modeler. The calculated model’s accuracy was 100% for classifying the patients into two groups depending on the number of adverse pregnancy outcomes (no APOs/at least one APO). The most important predictors of this model were a SLEDAI-2k score ≥4 from the first trimester, maternal body mass index, use of low-dose aspirin, proteinuria present in the third trimester, low complement (C3) in the first and second trimesters, decreased cerebroplacental ratio in the second trimester, personal history of lupus nephritis, hepatic cytolysis, and increased uric acid (Figure 1).

## 4. Discussion

The present study demonstrated a strong association between several predictors from the first, second, and third trimesters and adverse pregnancy outcomes.

The results of our study indicated that a BMI > 25 kg/m^2^ in pregnant SLE patients was significantly associated with adverse pregnancy outcomes. As for patient medical history, we found that lupus nephritis and chronic hypertension were factors with significant impact on pregnancy outcome.

Serial ultrasound evaluation of pregnant patients diagnosed with SLE is of utmost importance for diagnosing early pregnancy complications. In our study, increased mean uterine arteries pulsatility index in the first and second trimesters, cerebroplacental ratio less than 1 in the second and third trimesters, and small fetal abdominal circumference in the third trimester were significantly associated with adverse pregnancy outcomes.

The presence of proteinuria and low complement (C3) throughout pregnancy, along with hepatic cytolysis, increased uric acid, and active urinary cast in the third trimester were the most significant laboratory parameters associated with adverse pregnancy outcomes.

A SLEDAI-2k score ≥4 and a PGA score ≥1 were linked to at least one adverse pregnancy outcome, regardless of the trimester of pregnancy. On the other hand, the use of pulse therapy, a maintenance regimen of glucocorticoids, and the administration of methyldopa and low-dose aspirin appeared to be protective against adverse pregnancy outcomes.

Our results are in accordance with literature data, and the most cited clinical factors that influence pregnancy course in patients diagnosed with SLE include: younger age at onset, low complement, high proteinuria, high SLEDAI-2k score, thrombocytopenia, and the presence of lupus anticoagulant [22,23,24,25]. Moreover, in a recent retrospective study, the authors concluded that high SLEDAI-2k score, proteinuria, and hypocomplementemia within 6 months before conception and during pregnancy were associated with adverse maternal outcomes [26].

Zhan et al. investigated in a retrospective study the predictive performance of umbilical artery Doppler regarding APOs in pregnant patients with SLE, and demonstrated that an increased pulsatility index, with a 0.77 cut-off, had a sensitivity of 90.9% and a specificity of 49.2% [27]. In our study, a cerebroplacental index lower than 1 (associated with increased resistance on umbilical artery) was significantly associated with APOs. In another study, the authors prospectively investigated the association between increased pulsatility index of uterine arteries and fetal growth restriction for patients diagnosed with SLE, and outlined the high positive predictive value of this sonographic marker (83%) [28].

It was reported that approximately 20% of SLE patients experience disease flares during pregnancy [29], and that the frequency of APOs such as fetal/neonatal death, preterm birth, and intrauterine growth restriction is increased during disease flares [12,30]. In our study, SLE flares were successfully treated with methylprednisolone pulse therapy, excepting two cases who succumbed due to multi-system organ failure and sepsis of unknown origin.

A retrospective study by Kalok et al. indicated that aspirin and hydroxychloroquine were protective against fetal loss and preeclampsia in patients diagnosed with SLE [31]. Although low-dose aspirin administrated since the first trimester of pregnancy was found to be protective against APOs in our study, we could not prove the efficacy of hydroxychloroquine, probably due to our small cohort of patients.

Although the link between neonatal lupus and the presence of anti-Ro and anti-La antibodies in maternal serum is well established in the literature [32,33,34], we did not diagnose this entity in the newborns included in our study. The fetal heart was evaluated by experienced obstetricians using serial ultrasounds at 2 weeks between 16 and 14 weeks of gestation, and then weekly until birth [35]. The presence of anti-Ro antibodies was confirmed in four patients, and that of anti-La antibodies in only two patients. Their newborns did not show signs of neonatal lupus, although it cannot be excluded due to the short follow-up period.

In our study, none of the APS specific laboratory markers (LAC, aCL, and aβ2GPI) were significantly different between the two groups. Double positivity for LAC and aCL (*n* = 2 patients), and for LAC and aβ2GPI (*n* = 3 patients), were not significantly associated with adverse pregnancy outcomes in our cohort. None of our patients had a triple-positive antibody profile. Indeed, a recent multicentric study suggested that triple positivity in antiphospholipid antibody carriers was associated with higher rate of pregnancy complications [36].

We also created a predictive model based on linear discriminant analysis, which indicated that SLEDAI-2k score ≥4 from the first trimester, maternal body mass index, use of aspirin, proteinuria present in the third trimester, low complement (C3) in the first and second trimesters, decreased cerebroplacental ratio in the second trimester, personal history of lupus nephritis, hepatic cytolysis, and increased uric acid weighted the most in the model classification process.

A recent large prospective study developed three models using logistic regression for the evaluation of clinical predictors of APOs. The authors concluded that pregnancy outcomes were highly favorable in the absence of baseline features indicative of risk (LAC positive, antihypertensive medications, PGA >1, Hispanic or non-White ethnicity/race, low platelet count) [37].

Recent studies explored genomic, proteomic, and metabolomic approaches for identifying predictive biomarkers of APO in pregnant patients diagnosed with SLE [32,38,39,40,41]. Whereas the results reported in the literature differ from one study to another, further research could allow the incorporation of highly effective markers into predictive models.

The findings of this study must be seen in light of some limitations: small cohort size, few biological predictors included, and lack of newborns’ long-term follow-up. Previous research has identified several risk factors as predictors of adverse pregnancy outcomes. Nonetheless, the current literature has limitations due to diverse study designs, case definitions, and management practices in different centers.

## 5. Conclusions

With an increasing understanding of risk factors that predict APOs in SLE pregnancy, obstetricians can create therapeutic strategies based on individual women’s risk profiles.

This study provides a comprehensive analysis of the most important predictors for the main observed adverse pregnancy outcomes, and could constitute a basis for further research.

Careful evaluation and monitoring of pregnant SLE patients, implementation of prevention strategies, and active obstetric management are key elements for a successful pregnancy outcome.

## Figures and Tables

**Table 1:** Characteristics of pregnant SLE patients in the first trimester, and their association with adverse pregnancy outcomes (APOs).

Patient Data		No APOs	At Least One APO	*p* Value
Demographics	Age (years, mean ± SD)	30.53 ± 4.29	26.9 ± 6.6	0.054
	Medium n (%)	Rural = 8 (61.5%) Urban = 7 (58.3%)	Rural = 5 (38.5%) Urban = 5 (41.7%)	0.87
	BMI (kg/m^2^, mean ± SD)	21.87 ± 2.47	26.4 ± 1.075	<0.001
Patient history	Venous thrombosis n (%)	Yes = 1 (50%) No = 14 (60.9%)	Yes = 1 (50%) No = 9 (39.1%)	0.763
	Recurrent pregnancy loss n (%)	Yes = 2 (50%) No = 13 (61.9%)	Yes = 2 (50%) No = 8 (38.1%)	0.656
	Lupus nephritis n (%)	Yes = 1 (20%) No = 14 (70%)	Yes = 4 (80%) No = 6 (30%)	0.041
	Maternal diabetes n (%)	Yes = 2 (40%) No = 13 (59.1%)	Yes = 3 (60%) No = 7 (40.9%)	0.307
	Thyroid disorder n (%)	Yes = 2 (66.7%) No = 13 (86.7%)	Yes = 1 (33.3%) No = 9 (90%)	0.802
	Chronic hypertension n (%)	Yes = 0 (0%) No = 15 (71.4%)	Yes = 4 (100%) No = 6 (28.6%)	0.008
Clinical and paraclinical data	Uterine artery PI (mean ± SD)	1.15 ± 0.2	1.58 ± 0.6	<0.001
	Thrombocytopenia n (%)	Yes = 4 (40%)	Yes = 6 (60%)	0.096
		No = 11 (73.3%)	No = 4 (26.7%)	
	Proteinuria n (%)	Yes = 2 (22.2%)	Yes = 7 (77.8%)	0.004
		No = 13 (81.3%)	No = 3 (18.8%)	
	Anti-dsDNA n (%)	Yes = 10 (62.5%)	Yes = 6 (37.5%)	0.734
		No = 5 (55.6%)	No = 4 (44.4%)	
	C3 n (%)	Normal = 13 (81.3%)	Normal = 3 (18.8%)	0.004
		Low = 2 (22.2%)	Low = 7 (77.8%)	
	C4 n (%)	Normal = 13 (65%)	Normal = 7 (35%)	0.307
		Low = 2 (40%)	Low = 3 (60%)	
	LAC n (%)	Negative = 11 (64.7%)	Negative = 6 (35.3%)	0.484
		Positive = 4 (50%)	Positive = 4 (50%)	
	aCL n (%)	Negative = 14 (60.8%)	Negative = 9 (39.1%)	0.763
		Positive = 1 (50%)	Positive = 1 (50%)	
	Anti-β2 glycoprotein-I antibodies n (%)	Negative = 13 (59.09%)	Negative = 9 (40.9%)	0.801
		Positive = 2 (66.6%)	Positive = 1 (33.3%)	
	Anti-Ro n (%)	Negative = 12 (57.1%)	Negative = 9 (42.9%)	0.504
		Positive = 3 (75%)	Positive = 1 (25%)	
	Anti-La n (%)	Negative = 14 (60.9%)	Negative = 9 (39.1%)	0.763
		Positive = 1 (50%)	Positive = 1 (50%)	
	SLEDAI-2k n (%)	≥4 = 3 (27.3%)	≥4 = 8 (72.7%)	0.003
		<4 = 12 (85.7%)	<4 = 2 (14.3%)	
	PGA n (%)	≥1 = 2 (20%)	≥1 = 8 (80%)	<0.001
		<1 = 13 (86.7%)	<1 = 2 (13.3%)	

**Table 2:** Characteristics of pregnant SLE patients in the second trimester, and their association with adverse pregnancy outcomes (APOs).

Patient Data		No APOs	At Least One APO	*p* Value
Clinical data	Uterine artery PI (mean ± SD)	1.21 ± 0.12	1.66 ± 0.59	<0.001
	Cerebroplacental ratio n (%)	<1 = 0 (0%)	<1 = 3 (100%)	0.024
		>1 = 15 (68.2%)	>1 = 7 (31.8%)	
	Thrombocytopenia n (%)	Yes = 4 (44.4%)	Yes = 5 (55.6%)	0.234
		No = 11 (68.8%)	No = 5 (31.3%)	
	Proteinuria n (%)	Yes = 4 (36.4%)	Yes = 7 (63.6%)	0.032
		No = 11 (78.6%)	No = 3 (21.4%)	
	Anti-dsDNA n (%)	Yes = 9 (64.3%)	Yes = 5 (35.7%)	0.622
		No = 6 (54.5%)	No = 5 (45.5%)	
	C3 n (%)	Normal = 12 (92.3%)	Normal = 1 (7.7%)	<0.001
		Low = 3 (25%)	Low = 9 (75%)	
	C4 n (%)	Normal = 12 (70.6%)	Normal = 5 (29.4%)	0.11
		Low = 3 (37.5%)	Low = 5 (62.5%)	
	SLEDAI-2k n (%)	≥4 = 2 (18.2%)	≥4 = 9 (81.8%)	<0.001
		<4 = 13 (92.9%)	<4 = 1 (7.1%)	
	PGA n (%)	≥1 = 1 (12.5%)	≥1 = 7 (87.5%)	<0.001
		<1 = 14 (82.4%)	<1 = 3 (17.6%)	

**Table 3:** Characteristics of pregnant SLE patients in the third trimester, and their association with adverse pregnancy outcomes (APOs).

Patient Data		No APOs	At Least One APO	*p* Value
Clinical data	Cerebroplacental ratio n (%)	<1 = 0 (0%)	<1 = 3 (100%)	0.024
		>1 = 15 (68.2%)	>1 = 7 (31.8%)	
	Venous duct n (%)	Abnormal = 0 (0%)	Abnormal = 2 (100%)	0.071
		Normal = 15 (65.2%)	Normal = 8 (34.8%)	
	Fetal abdominal circumference (<10th percentile) n (%)	Normal = 15 (93.8%)	Normal = 1 (6.3%)	<0.001
		Low = 0 (0%)	Low = 9 (100%)	
	Anemia n (%)	Yes = 5 (71.4%)	Yes = 2 (28.6%)	0.467
		No = 10 (55.6%)	No = 8 (44.4%)	
	Leukopenia n (%)	Yes = 1 (50%)	Yes = 1 (50%)	0.763
		No = 14 (60.9%)	No = 9 (39.1%)	
	Thrombocytopenia n (%)	Yes = 4 (44.4%)	Yes = 5 (55.6%)	0.234
		No = 11 (68.8%)	No = 5 (31.3%)	
	Hepatic cytolysis n (%)	Yes = 0 (0%)	Yes = 3 (100%)	0.024
		No = 15 (68.2%)	No = 7 (31.8%)	
	Proteinuria n (%)	Yes = 6 (40%)	Yes = 9 (60%)	0.012
		No = 9 (90%)	No = 1 (10%)	
	Active urinary cast n (%)	Yes = 1 (20%)	Yes = 4 (80%)	0.041
		No = 14 (70%)	No = 6 (30%)	
	Uric acid n (%)	High = 1 (12.5%)	High = 7 (87.5%)	<0.001
		Normal = 14 (82.4%)	Normal = 3 (17.6%)	
	Anti-dsDNA n (%)	Yes = 7 (58.3%)	Yes = 5 (41.7%)	0.87
		No = 8 (61.5%)	No = 5 (38.5%)	
	C3 n (%)	Normal = 13 (86.7%)	Normal = 2 (13.3%)	<0.001
		Low = 2 (20%)	Low = 8 (80%)	
	C4 n (%)	Normal = 11 (73.3%)	Normal = 4 (26.7%)	0.096
		Low = 4 (40%)	Low = 6 (60%)	
	SLEDAI-2k n (%)	≥4 = 13 (76.5%)	≥4 = 4 (23.5%)	0.014
		<4 = 2 (25%)	<4 = 6 (75%)	
	PGA n (%)	≥1 = 4 (36.4%)	≥1 = 7 (63.6%)	0.032
		<1 = 11 (78.6%)	<1 = 3 (21.4%)	

## Data Availability

The data presented in this study are available on request from the corresponding author. The data are not publicly available due to local policies.

## Supplementary Materials

The following supporting information can be downloaded at: https://www.mdpi.com/article/10.3390/jcm11071964/s1, Table S1. Maternal-fetal complications in our cohort of pregnant patients affected by SLE; Table S2. Summary of the main parameters taken into consideration for evaluation of pregnant patients diagnosed with SLE.
