# Supplementary material for: Predictors Associated with Adverse Pregnancy Outcomes in a Cohort of Women with Systematic Lupus Erythematosus from Romania—An Observational Study (Stage 2)
Source: J Clin Med. 2022 Apr 1;11(7):1964. doi: 10.3390/jcm11071964 (PMC9000014; doi:10.3390/jcm11071964)
Supplement: Supplementary file 1 [file jcm-11-01964-s001.zip › Table S1.pdf]

Table S1. Maternal-fetal complications in our cohort of pregnant patients affected by SLE.

Type of maternal and fetal complications	
SLE flares	**Renal**<br>First trimester- No<br>Second trimester-Yes (n=5)<br>Third trimester- Yes (n=5)<br>**Musculoskeletal**<br>First trimester- No<br>Second trimester-Yes (n=1)<br>Third trimester- Yes (n=4)
Preeclampsia (≥ 20 weeks of gestation)	Third trimester (n=3)
IUGR	Third trimester (n=3)
Preterm birth (before 37 weeks of gestation)	Yes (n=3)
Maternal death	Yes (n=2)
Neonatal death	None
Apgar score less than 7	None
Neonatal CHB	None
