# Supplementary material for: Predictors Associated with Adverse Pregnancy Outcomes in a Cohort of Women with Systematic Lupus Erythematosus from Romania—An Observational Study (Stage 2)
Source: J Clin Med. 2022 Apr 1;11(7):1964. doi: 10.3390/jcm11071964 (PMC9000014; doi:10.3390/jcm11071964)
Supplement: Supplementary file 1 [file jcm-11-01964-s001.zip › Table S2.pdf]

Table S2. Summary of the main parameters taken into consideration for evaluation of pregnant patients diagnosed with SLE.

First trimester	Second trimester	Third trimester
**Demographics**	**Clinical data**	**Clinical data**
		Cerebroplacental
Age	Uterine artery PI	ratio
Medium	Cerebro-placental	Venous duct
BMI	ratio	Fetal abdominal
	Thrombocytopenia	circumference
**Patient's history**	Proteinuria	(<10th percentile)
	Anti-dsDNA	Anemia
Venous thrombosis	C3	Leukopenia
Recurrent pregnancy loss	C4	Thrombocytopeni
Lupus nephritis	SLEDAI-2k	a
Maternal diabetes	PGA	Hepatic cytolysis
Thyroid disorder		Proteinuria
Chronic hypertension		Active urinary
		cast
		Uric acid
		Anti-dsDNA
		C3
		C4
		SLEDAI-2k
**Clinical and paraclinical data**		PGA
Uterine artery PI		
Thrombocytopenia		
Proteinuria		
Anti-dsDNA		
C3		
C4		
LAC		
aCL		
Anti-β2 glycoprotein-I anti-bodies		
Anti-Ro		
Anti-La		
SLEDAI-2k		
PGA		
